# Identification of Continuous Human B-Cell Epitopes in the VP35, VP40, Nucleoprotein and Glycoprotein of Ebola Virus

**DOI:** 10.1371/journal.pone.0096360

**Published:** 2014-06-10

**Authors:** Pierre Becquart, Tanel Mahlakõiv, Dieudonné Nkoghe, Eric M. Leroy

**Affiliations:** 1 UMR 264 MIVEGEC, Institut de Recherche pour le Développement (IRD). Montpellier, France; 2 Centre International de Recherches Médicales de Franceville, Franceville, Gabon; National Institutes of Health. National Institute of Allergy and Infectious Diseases, Division of Clinical Research, United States of America

## Abstract

Ebola virus (EBOV) is a highly virulent human pathogen. Recovery of infected patients is associated with efficient EBOV-specific immunoglobulin G (IgG) responses, whereas fatal outcome is associated with defective humoral immunity. As B-cell epitopes on EBOV are poorly defined, we sought to identify specific epitopes in four EBOV proteins (Glycoprotein (GP), Nucleoprotein (NP), and matrix Viral Protein (VP)40 and VP35). For the first time, we tested EBOV IgG+ sera from asymptomatic individuals and symptomatic Gabonese survivors, collected during the early humoral response (seven days after the end of symptoms) and the late memory phase (7–12 years post-infection). We also tested sera from EBOV-seropositive patients who had never had clinical signs of hemorrhagic fever or who lived in non-epidemic areas (asymptomatic subjects). We found that serum from asymptomatic individuals was more strongly reactive to VP40 peptides than to GP, NP or VP35. Interestingly, anti-EBOV IgG from asymptomatic patients targeted three immunodominant regions of VP40 reported to play a crucial role in virus assembly and budding. In contrast, serum from most survivors of the three outbreaks, collected a few days after the end of symptoms, reacted mainly with GP peptides. However, in asymptomatic subjects the longest immunodominant domains were identified in GP, and analysis of the GP crystal structure revealed that these domains covered a larger surface area of the chalice bowl formed by three GP_1_ subunits. The B-cell epitopes we identified in the EBOV VP35, VP40, NP and GP proteins may represent important tools for understanding the humoral response to this virus and for developing new antibody-based therapeutics or detection methods.

## Introduction

Ebola virus (EBOV), a member of the *Filoviridae* family, is a highly virulent pathogen for humans and nonhuman primates [1]. EBOV is a filamentous enveloped virus containing a negative-strand RNA genome of about 19 kb. The EBOV genome codes for eight major subgenomic mRNAs that sequentially encode seven structural proteins, namely a nucleoprotein (NP), two virion proteins (VP35 and VP40), a surface glycoprotein (GP), two additional viral proteins (VP30 and VP24), an RNA-dependent RNA polymerase (L), and a non-structural soluble protein (sGP) [2].

After an incubation period ranging from 2 to 21 days (mean 4–9 days), EBOV causes severe hemorrhagic fever that is fatal in nearly 90% of cases within 7–11 days [3]. EBOV has caused several outbreaks in Gabon, Democratic Republic of the Congo (DRC) and Republic of the Congo (RC) [4]–[7]. There is currently no vaccine and no specific treatment. Fatal EBOV infection is characterized by a defective innate immune response, leading to uncontrolled release of inflammatory mediators and chemokines in the late stage of the disease, and correlates with the collapse of adaptive immunity with massive T and B lymphocyte apoptosis [8]–[10]. However, lethally infected model mice generated a functional CD8+ T cell response despite significant T cell apoptosis [11]. Survivors and asymptomatic subjects develop an early and moderate inflammatory response together with an effective adaptive response [8]–[10]. Defective adaptive immunity observed in fatal cases is associated with an impaired humoral response, as EBOV-specific IgG and IgM are barely detectable before death [12]–[14]. In contrast, recovery is associated with early, increasing levels of long-lasting EBOV-specific IgG, followed by viral antigen clearance [12], [15], [16]. Moderate amounts of EBOV-specific IgG are also detected about 3 weeks after infection in asymptomatic patients [8]. A strong early humoral response may thus play a major role in survival.

Little is known of human antibody targets in EBOV infection. Western blot analysis has shown that IgG antibodies in sera from survivors of symptomatic infection and from asymptomatic subjects are mainly directed against NP and VP40, and, in a minority of cases, against VP35 [8], [12]. Similar results have been reported in seropositive individuals who have never had clinical signs of hemorrhagic fever or who live in non-epidemic areas [17]. Antibody phage display libraries constructed from RNA derived from two survivors of the 1995 EBOV outbreak in Kikwit (DRC) also showed the presence of antibodies reacting with NP, GP and sGP [18]. Little further information is available on human B-cell epitopes of EBOV proteins. Thus, the purpose of the present study was to identify immunodominant IgG-specific epitopes in GP, NP, VP40 and VP35 using, for the first time, anti-EBOV IgG+ patient sera.

## Materials and Methods

### B cell epitope mapping

In order to identify linear epitopes and to characterize immunodominant sites in EBOV, we applied epitope mapping to four EBOV proteins representing the main targets of the humoral response, namely VP35, VP40, NP and GP. Five hundred and six peptides (consecutive 15-mers with an overlap of 11 aminoacids (aa) were synthesized by Eurogentec (Belgium). The peptides spanned the 676 aa of GP (162 peptides), the 739 aa of NP (182 peptides), the 326 aa of VP40 (79 peptides) and the 340 aa of VP35 (83 peptides), as deduced from the EBOV genome isolated during the 1995 outbreak in Kikwit, DRC [19]. Each lyophilized peptide was solubilized in dimethyl formamide and adjusted to a final concentration of 5 mg/mL with phosphate buffered saline (PBS) before use (Sigma, France).

### Ebola patient sera

**Figure 1:**
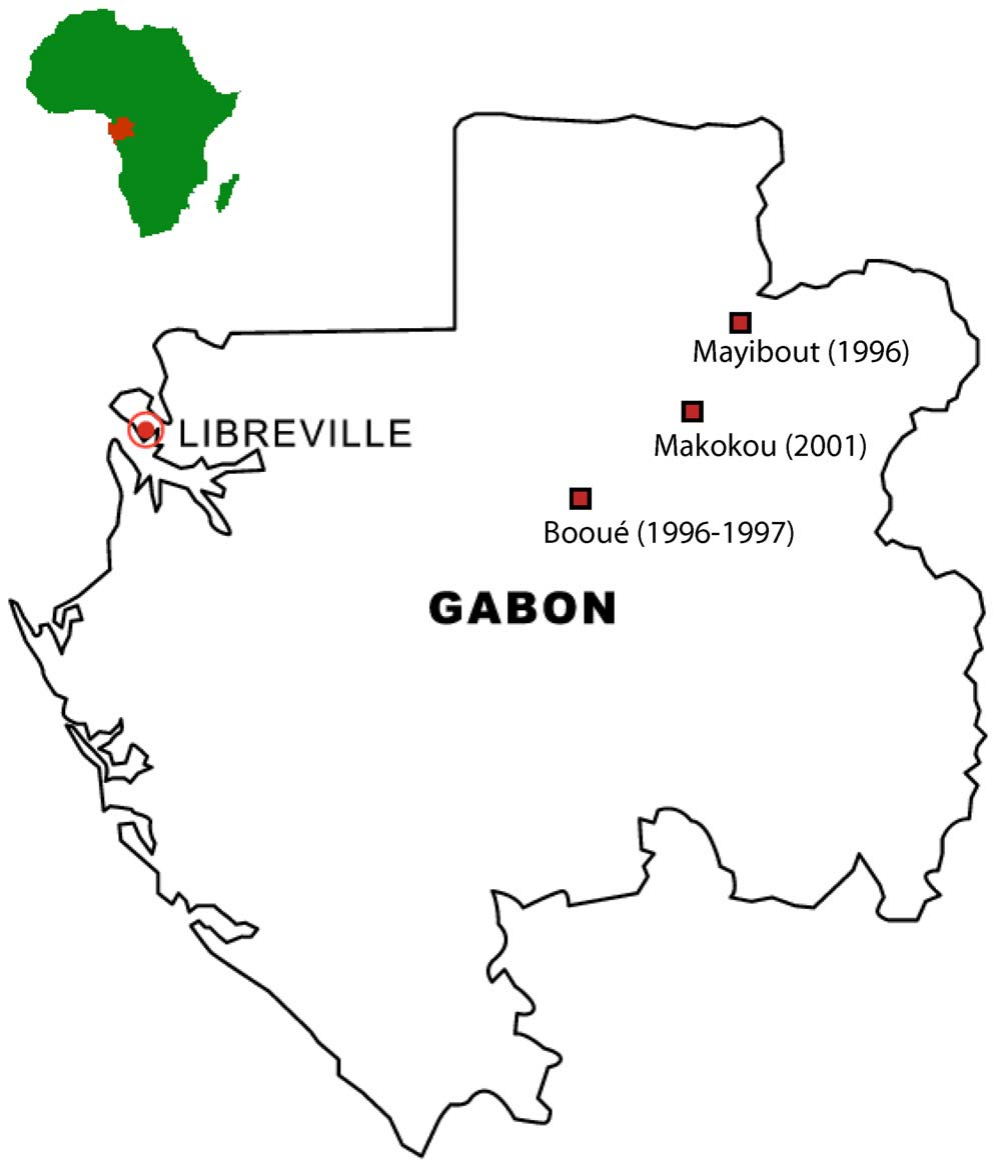
Map of Gabonese villages where the 1996 and 2001 outbreaks occurred.

We used serum samples from EBOV-infected patients collected during the outbreaks in the villages of Mayibout, Booué (1996) and Mekambo (2001) in Gabon (Figure 1). The first outbreak hit the village of Mayibout, located in north-eastern Gabon, from January to February 1996, causing 10 non-fatal clinical cases and 21 deaths [6]. The second outbreak caused 45 deaths among 60 cases between October 1996 and March 1997 in the Booué area, 150 km southwest of Mayibout [6]. The infection spread to several villages around Booué. The third outbreak occurred between October 2001 and May 2002 in the Mekambo area of Gabon and the Mbomo area of Republic of Congo, 150 km east of Mayibout [20]. A total of 207 human cases (58 survivors and 149 deaths) were recorded. A total of 298 human cases and 215 deaths were reported during these three outbreaks. Sera from two groups of survivors were used to map B cell epitopes. The first consisted of 15 stored sera collected during the three outbreaks from survivors of laboratory-confirmed infection, seven days after the end of symptoms (Survivor group #1) [8], [12]. The second (Survivor group #2) consisted of sera collected in 2008 from a survivor of the 1996 Mayibout outbreak, two survivors of the 1996–1997 Booué outbreak and three survivors of the 2001 Mekambo outbreak [15]. Early humoral responses to EBOV were analyzed in the first survivor group, and late humoral responses were analyzed in the second group. All the individuals were adults (>16 years), except for patient #19 who was 7 years old and patients #1 and #2 who were 15 years old. We also screened 21 sera from EBOV IgG-seropositive adults who had never had clinical signs of hemorrhagic fever or who lived in non-epidemic areas. These individuals, selected randomly in various parts of Gabon, had tested positive for anti-EBOV antibodies in an indirect IgG ELISA and by western blot [17]. They lived in rural villages with fewer than 300 inhabitants, located in the nine administrative regions of Gabon. As negative controls, we randomly selected 20 individuals from Gabon who had tested negative for EBOV by indirect ELISA and western blot methods, as described elsewhere [13], [17].

Sera from each group were pooled for the first screen with all the synthetic peptides. The first two pools consisted of sera from eight and seven survivors collected 7 days after the end of symptoms (Survivor group #1). The third pool consisted of six stored sera from survivors collected in 2008 (Survivor group #2). The last three pools each consisted of sera from seven asymptomatic individuals. Two pools of sera from ten EBOV-naive individuals were used as negative controls.

### Ethics statement

Our study received the approval of the Gabonese Ministry of Health (research authorization Nb. 00093, March 15, 2005). Samples from the present study came from two previous serosurveys conducted by a multidisciplinary team, including a doctor from the Gabonese Ministry of Health, a nurse, an epidemiologist, a virologist, a veterinarian and laboratory technicians [15], [17]. The studies were approved by the traditional chief of each village, and written informed consent was obtained from each survivor and asymptomatic patient. The studies were described orally, and volunteers gave their signed informed consent to be enrolled in the studies and for their blood samples to be used for future research studies. In the case of children, the parents' written consent was obtained for their clinical records and samples to be used for future research studies.

### Indirect ELISA for peptide epitope identification

Briefly, plates (Nunc-immuno Maxisorb plates, Nunc, Denmark) were coated with 5 µg/mL single synthetic peptide in 100 µL/well PBS overnight at +4°C. The wells were then washed three times with PBS containing 0.1% (vol/vol) Tween 20 (Sigma) (PBS-T). To block non-specific binding, the wells were incubated with 200 µL of PBS-T containing 5% (weight/vol) non-fat milk (Becton Dickinson) (mPBS-T) for 1 hour at 37°C. Based on our unpublished data, we tested several serum dilutions (1∶100 to 1∶1600) for each peptide. The 1∶400 dilution provided the best compromise between sensitivity and specificity in our ELISA. Therefore, after the washing step described above, 1∶400-diluted sera in mPBS-T were incubated in the wells overnight at +4°C. The wells were then washed three times with PBS-T and 100 µL of goat anti-human IgG peroxidase-labeled antibody (Sigma) was added at 1∶2000 dilution in mPBS-T for 1 hour at room temperature. After a final washing step with PBS-T, 100 µL of a 1∶1 solution of tetramethylbenzydine (TMB, Thermo Electron Corporation) and hydrogen peroxide (Dynex Technologies, France) was added to each well for 10 minutes at room temperature. Optical density (OD) was then measured at 450 nm with an ELISA plate reader (Bio-Rad PR3100).

All 506 peptides provided by Eurogentec were tested in triplicate against the positive and negative serum pools as described above. The mean OD obtained with the negative serum pool, plus three times the standard deviation, was used as the cut-off value. The reactive peptides identified in the first screen were then tested in triplicate with individual sera from the 21 survivors, the 21 positive asymptomatic seropositive subjects, and the 20 EBOV-naive individuals. The same cut-off as above was used for each reactive peptide selected in the first screen. Final results are reported as mean OD.

## Results

### Epitope mapping of VP35, VP40, NP and GP

#### 1. Identification and location of reactive peptides in survivors

**Figure 2:**
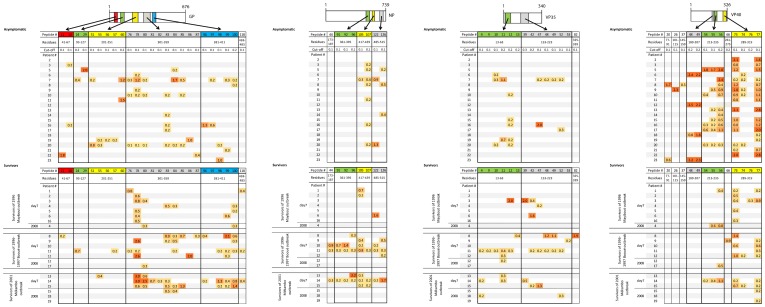
Mean OD values at 1∶400 serum dilution in patients infected with EBOV during three outbreaks in Gabon, 7 days after symptom onset (Day 7, early humoral response) and 7 or 11 years later (2008, late humoral response), and also in anti-EBOV IgG+ asymptomatic individuals who had never had clinical signs of hemorrhagic fever or who lived in non epidemic areas*. ODs are color-coded to indicate their intensity (yellow to red). The different colored regions in the proteins indicate the immunodominant domains identified here. *Blank cells indicate that the mean OD was lower than the cut-off. OD: Optical Density. The absorbance cut-off used to identify reactive epitopes is described in the Methodology section.

**Figure 3:**
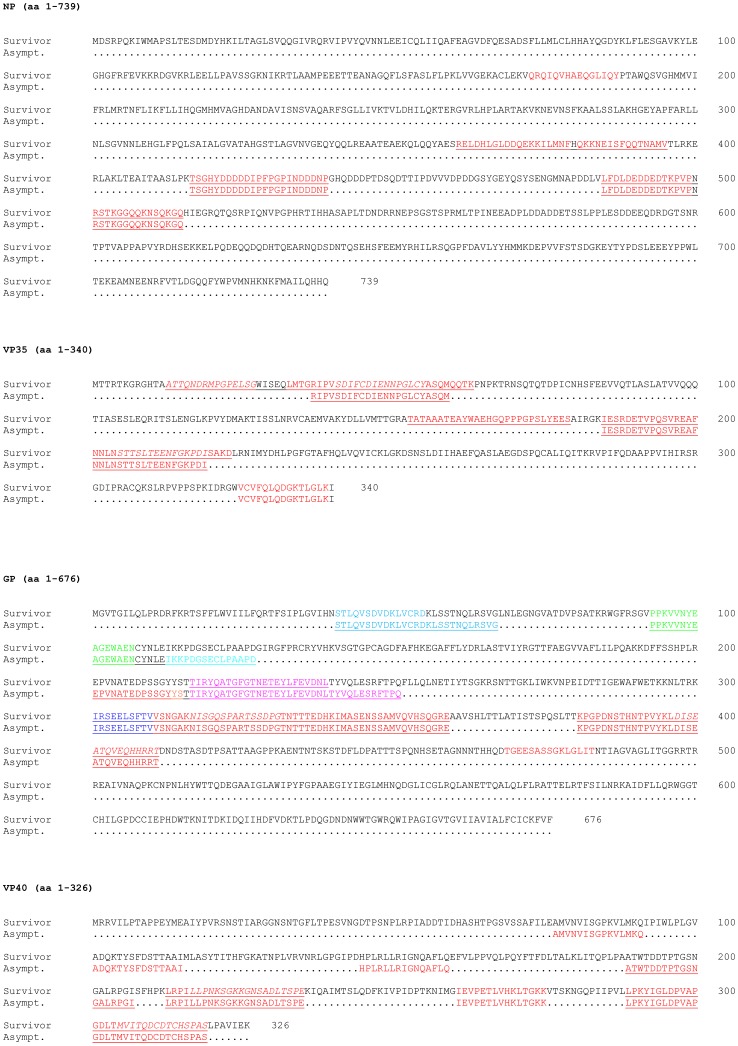
Epitopes reacting with sera collected from survivors of the three outbreaks in Gabon, 7 days after symptom onset, and from asymptomatic individuals (Asympt.) who had never had clinical signs of hemorrhagic fever or who lived in non epidemic areas, are indicated in color. GP epitopes shown in different colors correspond to the regions of the GP protein structure in Figure 4. Epitopes reacting with sera from survivors of the three outbreaks, collected 7 or 11 years after recovery, are indicated in italics. The principal immunogenic regions are underlined.

**Figure 4:**
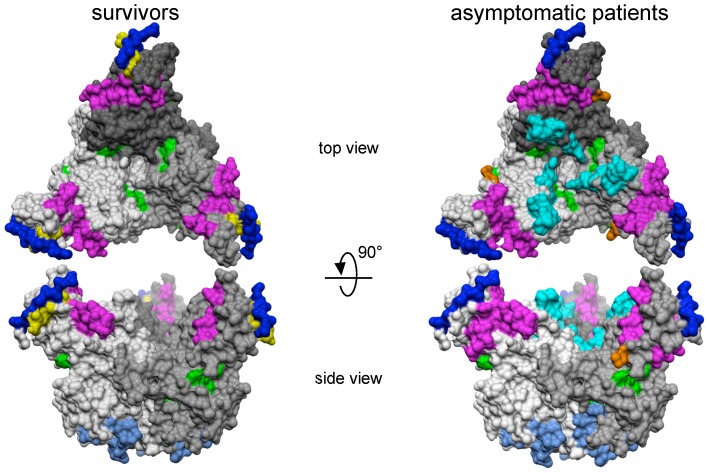
Crystal structure of the trimeric prefusion EBOV GP viewed from the top (above) and side (below) [21]. The three monomers in the complex are colored different shades of grey. Immunodominant regions identified in GP are indicated in color for survivors and asymptomatic patients. The color code matches that of the epitopes identified in Figure 3. Molecular surface of the GP trimer viewed from the side (left) and top (right), as viewed down the three-fold axis. Protein Data Bank file number: 3CSY.

In the first screen, the two pools of sera from survivors collected 7 days after the end of symptoms (Survivor group #1) and from survivors collected in 2008 (Survivor group #2) reacted with 14 VP35 peptides, 8 NP peptides, 7 VP40 peptides, and 19 GP peptides. In the second screen, sera from the 21 survivors were tested with these reactive peptides. The sequences of these peptides and the mean ODs are summarized in Figure 2. The humoral response was mainly directed against GP. None of the peptides was recognized by all of the survivor group #1 and #2 sera, but GP peptide 78, NP peptide 105 and VP40 peptides 73 and 77 reacted with more than half of the group #1 sera. These peptides composed several linear regions which were similar during the three EBOV outbreaks that occurred in Gabon between 1996 and 2001. We thus identified 10 immunogenic regions in the four proteins tested (Figure 3). The immunodominant epitopes recognized by anti-EBOV IgG were located in three principal regions of NP and GP at aa positions 361–395, 417–439, 485–515 and 216–239, 301–359, 381–411 respectively. Two antigenic regions were mapped in VP40 (213–235 and 289–319) and VP35 (13–63 and 153–223). Sera of survivor groups #1 and #2 reacted with GP, VP40, NP and VP35 peptides in 71%, 100%, 38% and 38% of cases, respectively. The crystal structure of the trimeric prefusion EBOV GP complexed with a neutralizing antibody from a human survivor [21] was used to locate GP immunodominant regions with the assistance of Cn3D 4.3 software (National Center for Biotechnology Information). The regions at aa positions 301–359 and 381–411 of the GP mucin-like domain could not be located, as no structural information is available for this domain and as GPs containing the heavily glycosylated mucin-like domain have been refractory to crystallization. In the crystal structure of GP lacking the mucin-like domain, survivors' IgG was mainly directed against epitopes located on the inner and outer surfaces of the chalice bowl formed by three GP_1_ viral attachment subunits, cradled by the GP_2_ fusion subunits (Figure 4).

#### 2. Identification and location of reactive peptides in asymptomatic subjects

Peptide ELISA was also used to screen sera from anti-EBOV IgG+ individuals who had never had clinical signs of hemorrhagic fever or who lived in non-epidemic areas, and in whom we had previously detected both EBOV-specific IgG and IFN-γ-producing CD8+ T cell responses [17]. In the first screen, the three pools, each containing sera from seven asymptomatic individuals, reacted with seven peptides located in VP35, 11 in VP40, four in NP and 22 in GP. In the second screen, sera from the 21 positive asymptomatic seropositive subjects were tested individually with these reactive peptides. The sequences of these peptides and their adjusted ODs are summarized in Figure 2. Most of the reactive peptides were located in 11 linear immunogenic regions at aa positions 37–59 and 153–219 for VP35, 417–439 and 485–515 for NP, 189–235 and 289–319 for VP40 and 41–67, 93–127, 201–251, 301–359 and 381–411 for GP (Figure 3). GP, VP40, NP and VP35 peptides reacted respectively with 81%, 76%, 52% and 57% of sera from asymptomatic individuals. In these subjects the main target of the anti-EBOV IgG response was VP40 (Figure 2). As in survivors, none of the peptides reacted with all the sera, but VP40 peptides 73 and 77 reacted with more than 70% of the sera from the 21 individuals (n = 15 and n = 16, respectively). The positions of the immunodominant regions in a three-dimensional structural projection of GP were located as described above. We found that the epitopes recognized by these asymptomatic subjects' sera were located in the chalice bowl formed by the three GP_1_ subunits (Figure 4).

#### 3. Comparison between early and late responses in survivors

Although the number of survivors of the 1996 and 2001 outbreaks available for sampling in 2008 was small (survivors #4, #12 and #15), the data suggests that the number of B-cell epitopes is far lower in sera collected seven to twelve years after infection than in sera collected during the acute phase (Figure 2). Indeed, EBOV peptide-reactive IgG was undetectable in sera collected more than 11 years after infection (Survivor group #2), compared to sera from individuals in early convalescence (Survivor group #1) (n = 23; 74.2%).

#### 4. Comparison between symptomatic and asymptomatic subjects

The panel of peptides recognized by sera from asymptomatic subjects was similar to that recognized by sera from the two groups of survivors (Figure 3). Proportionally, the IgG response in survivors was mainly directed against GP, while in asymptomatic subjects it was mainly directed against VP40. In addition, more VP40 immunogenic regions were found in asymptomatic patients than in survivors (7 *versus* 3). Interestingly, in the asymptomatic subjects the longest immunodominant domains were identified in GP_1_, and the crystal structure of GP revealed that these regions covered a larger surface area of the chalice bowl formed by the three GP_1_ subunits (Figure 4).

## Discussion

Multiple anti-EBOV monoclonal antibodies, including neutralizing antibodies, have been generated, and epitopes, mainly located in the viral glycoprotein, have been identified in rodent models [22], [23]. A recent study showed that passive transfer of species-matched polyclonal IgG, obtained from a large quantity of convalescent serum from surviving vaccinated rhesus macaques challenged with EBOV and Marburg virus, provided complete protection from filovirus infection in non human primates [24]. Little is known, however, of the targets of the human humoral response to EBOV. Here, for the first time, we identified such epitopes in GP, NP, VP40 and VP35, the main antibody targets in EBOV-infected patients. Sera from asymptomatic subjects and survivors were tested against a set of overlapping 15-residue synthetic peptides that spanned the four proteins.

We found that antisera from asymptomatic patients targeted and reacted strongly with a larger number of peptides derived from VP40 than from GP, NP or VP35. This confirmed our earlier results obtained by western blotting in the same individuals [17]. In addition, a previous western blot analysis of sera collected from asymptomatic patients revealed that IgG responses were directed against both VP40 and NP [8]. The structural protein VP40 is the most abundant protein in EBOV particles, representing about 40% by molecular weight [25]. The matrix proteins VP40 and VP24 are linked to the ribonucleoprotein complex at the inner surface of the lipid bilayer of the viral envelope (which is derived from the host cell), ensuring the structural integrity of the particle [25], [26]. VP40 plays a crucial role in virus assembly and budding [27], [28]. This matrix protein is able to produce virus-like particles (VLPs) from cells in the absence of other viral proteins [26], [29], [30]. Dessen *et al.* first elucidated its crystal structure and suggested that the protein consists of two distinct N- and C-terminal domains (NTD and CTD) connected by a flexible linker [31], [32], both essential for trafficking to and interaction with the membrane [27], [33]. More recent crystal structures reveal that VP40 is a dimer, assembling end-to-end into filaments, through homologous interactions between their CTDs [34]. Here, we found that serum from asymptomatic individuals reacted strongly with three immunodominant domains located in the CTD of VP40 (aa residues 189–207, 213–235 and 289–319), while serum from survivors reacted less strongly and with fewer peptides. The first region, targeted only by IgG from asymptomatic patients, is located in the flexible linker (residues 195–200) connecting the structurally similar beta-sandwiches of CTD and NTD [31]. The second region is located in a conserved basic patch essential for electrostatic membrane interaction and for triggering assembly of VP40 into the viral matrix to bud nascent virions [34], [35]. The third region is located in the hydrophobic dimeric VP40 CTD-to-CTD interface, which has been shown to be critical for matrix assembly and budding [34]. Residue 307 appears to be particularly important, as the RNA-binding structures formed by the VP40-I307R mutant do not migrate to the membrane, assemble the viral matrix, or bud VLPs. However, the majority of VP40 is located inside the virion and not exposed to the outer surface, a very recent study indicates its importance in inducing immune responses [36]. Taken together, our findings strongly suggest that the humoral responses directed against these three key epitopes of VP40 may contribute to protecting humans against EBOV infection. The protective efficacy of the humoral response could be further evaluated by using functional test such as seroneutralization and/or Antibody-Dependant Cellular Cytotoxicity (ADCC) assays.

We also found that serum from most survivors of the three outbreaks, collected a few days after the end of symptoms, reacted mainly with GP peptides. This protein is organized as trimeric spikes consisting of two subunits, namely the extracellular subunit GP_1_ attached to the membrane-anchored subunit GP_2_ [21]. GP_1_ is responsible for cell surface attachment, while GP_2_ is required for fusion of the viral and host cell membranes [37]–[39]. GP is the only viral protein expressed on the surface of infected cells and could thus be an ideal target for neutralizing antibodies. A number of studies have assessed the efficacy of EBOV GP-specific neutralizing antibodies as post-exposure therapeutics in animal models, but with variable results [22], [40]–[45]. A recent study showed that passive transfer of a mixture of two human-mouse chimeric neutralizing mAbs directed against GP conferred partial protection in rhesus macaques challenged with EBOV [46]. Our ELISA studies showed that reactive GP peptides were distributed in five immunogenic regions located at aa positions 41–67, 93–127, 201–251, 301–359 and 381–411. Two of these regions belong to a domain located in the C-terminal portion of GP_1_, which contains a highly N- and O-glycosylated mucin-like domain. This domain is extremely large and represents more than half the molecular weight of GP [21]. Neutralizing antibodies reacting with the mucin-like domain have been shown to protect mice from lethal EBOV infection [22]. Interestingly, the immunogenic region at aa position 381–411 and the antigenic peptide #118 (aa position 469–483) highlighted in our study covers three linear epitopes (aa positions 401–417, 389–405 and 477–493) recognized by these protective antibodies. In addition, our results reveal a new, larger IgG-specific immunodominant region in the mucin-like domain of GP, located at the beginning of the domain protein sequence (aa residues 301 to 359). As GP and sGP share 295 aa in their N-terminal sequences and have several epitopes in common [47], [48], neutralizing antibodies produced during natural infection appear to react preferentially with sGP, which is abundantly shed by infected cells in acutely infected patients [18]. sGP, the pathogenic role of which is unclear, appears to act as a decoy for GP-specific neutralizing antibodies [18], [22]. However, the mucin-like domain is specific to GP, suggesting that neutralizing antibodies reacting with this domain would be more effective for the treatment of EBOV infection than those that react with GP and sGP, which would likely be bound by the much more abundant sGP. Unfortunately, no structural information on the mucin-like domain is available. However, the crystal structure of the truncated region of this protein suggests that the mucin-like domain extends from the top and sides of GP and effectively dominates its structure, being in a position suitable for host interaction or for protecting the rest of the GP protein from immune recognition [21]. In addition, the heavy glycosylation of this domain could play a role in immune evasion by masking antibody epitopes [49]–[52]. Interestingly, a previous study showed that the epitopes of the mucin-like domain recognized by two neutralizing and protective antibodies were not glycosylated [53]. The mucin-like domain does not appear to have a critical function in GP-mediated cell entry or membrane fusion, but it has been implicated in EBOV GP-induced cytotoxicity *in vitro* and has been shown to affect cell adhesion [37], [54]–[57]. It has also been reported that this domain is sufficient to cause GP-characteristic cytopathology [58], while its deletion reduced immunogenicity in a mouse model [59]. Although the sequence of this region is different in the five EBOV species, selective pressure appears to have conserved both its overall length and its level of glycosylation, suggesting an important role in filovirus pathogenesis. The other three immunogenic regions we identified in GP were located in GP_1_, being situated on the chalice of the viral surface trimer formed by three GP_1_ subunits, that are encircled by three GP_2_ subunits forming a cradle. Interestingly, we found that the IgG response encompassed a larger surface area of the chalice bowl in asymptomatic subjects than in survivors. Intriguingly, two of these three immunogenic regions were located in a putative binding site for attachment to host cells, including residues 54–201 [56], [60], [61], which are sequestered in the chalice bowl of GP_1_ [21]. The crystal structure of EBOV GP also suggests that the mucin-like domain restricts access to the conserved receptor-binding site. The regions harboring reactive peptides in GP_1_ identified here could be relevant for the development of EBOV-protective mAbs.

Interestingly, two immunogenic regions at aa positions 417–439 and 485–515 highlighted in our study cover linear epitopes recognized by mouse mAbs [62].

In previous studies, long-lasting IgG antibody responses were characterized by ELISA based on EBOV antigens extracted from tissue-culture cells infected with virus from survivors of EBOV outbreaks in Gabon and RC [13], [15], [63]. Persistent immune responses were also observed in human survivors by means of an ELISA based on full-length recombinant viral proteins of Sudan virus [64], [65]. However, we found that the number of peptides recognized by survivors' sera was significantly lower in sera collected more than ten years after infection than in sera collected during early convalescence. Differences in the ELISA methods used for EBOV-specific IgG detection could explain this discrepancy. Indeed, overlapping peptide ELISA allows the identification of linear epitopes [66], while the use of viral antigens also identifies antibodies targeting conformational epitopes [67], [68]. As the number of survivors of the 1996 and 2001 outbreaks available for sampling in 2008 was small (n = 3), our results showing a loss of EBOV-specific IgG several years after infection need to be confirmed. Moreover, it is known that virus-specific IgG antibody titers decrease with time after infection or vaccination [69]. Additionally, other infections, bacterial or viral, could play a role in these declining IgG titers [70]. Seroreversion has also been observed after Ebola infection [8], [10], [15].

Nucleotide sequence variations have been identified between EBOV strains from different epidemic chains, and phylogenetic analysis of the GP and NP sequences revealed that the Booué and Mayibout EBOV variants belonged to a lineage different from that of the Mekambo variant (lineage group A *versus* recombinant group R, respectively) [7], [71]–[73]. In the present study, the peptides reacting with sera from survivors of the three outbreaks were located in similar immunodominant IgG-specific regions, suggesting that the humoral response targets similar epitopes regardless of the EBOV strain.

## Conclusions

We show that the humoral response of survivors of Gabonese EBOV outbreaks mainly targeted GP peptides, whereas sera from EBOV-seropositive asymptomatic subjects reacted strongly with VP40 peptides. The B-cell epitopes we identified in the VP35, VP40, NP and GP proteins could be relevant for the development of new therapeutics or diagnostic tools and may represent alternatives to EBOV antigens for use in direct ELISA methods.
